# Isoflavone Content of Soybean Cultivars from Maturity Group 0 to VI Grown in Northern and Southern China

**DOI:** 10.1007/s11746-014-2440-3

**Published:** 2014-03-21

**Authors:** Jingying Zhang, Yinan Ge, Fenxia Han, Bin Li, Shurong Yan, Junming Sun, Lianzheng Wang

**Affiliations:** MOA Key Laboratory of Soybean Biology (Beijing), Institute of Crop Science, Chinese Academy of Agricultural Sciences, Beijing, 100081 People’s Republic of China

**Keywords:** Soybean (*Glycine max* L. Merrill), Isoflavone, Fatty acid, Protein, Yield, Maturity group

## Abstract

Soybean isoflavone content has long been considered to be a desirable trait to target in selection programs for their contribution to human health and plant defense systems. The objective of this study was to determine isoflavone concentrations of various soybean cultivars from maturity groups 0 to VI grown in various environments and to analyze their relationship to other important seed characters. Forty soybean cultivars were grown in replicated trials at Wuhan and Beijing of China in 2009/2010 and their individual and total isoflavone concentrations were determined by HPLC. Their yield and quality traits were also concurrently analyzed. The isoflavone components had abundant genetic variation in soybean seed, with a range of coefficient variation from 45.01% to 69.61%. Moreover, individual and total isoflavone concentrations were significantly affected by cultivar, maturity group, site and year. Total isoflavone concentration ranged from 551.15 to 7584.07 μg g^−1^, and averaged 2972.64 μg g^−1^ across environments and cultivars. There was a similar trend regarding the isoflavone contents, in which a lower isoflavone concentration was generally presented in early rather than late maturing soybean cultivars. In spite of significant cultivar × year × site interactions, cultivars with consistently high or low isoflavone concentrations across environments were identified, indicating that a genetic factor plays the most important role for isoflavone accumulation. The total isoflavone concentration had significant positive correlations with plant height, effective branches, pods per plant, seeds per plant, linoleic acid and linolenic acid, while significant negative correlations with oleic acid and oil content, indicating that isoflavone concentration can be predicted as being associated with other desirable seed characteristics.

## Introduction

Soybean isoflavone is an important secondary metabolite accumulated in soybean. A total of 12 types of component can be divided into four groups including free, glucoside, acetyl-glucoside and malonyl-glucoside forms [1]. Isoflavones have attracted much attention because of their important physiological functions in the prevention and treatment of cancer, cardiovascular disease, osteoporosis and senile dementia. Isoflavones also play important roles in anti-fungal, anti-tumor, antioxidant properties and reduction in women’s menopausal syndrome [2–4].

Isoflavone content is highly variable and regulated by genetic and environmental factors, and the qualitative-quantitative genetic character is controlled by several major and minor genes [5–8]. Significant differences in isoflavone content of different soybean varieties indicated the existence of genetic differences. Even the isoflavone content of one genotype variety planted at the same location demonstrated a change up to threefold in different years [9, 10]. Therefore, for isoflavone content, the yearly difference is more important than location and other environmental factors [11]. In addition to the year factor, other environmental factors are also involved in isoflavone contents and components. The malonyl-glucoside type is easily transformed to the corresponding aglycone form under high temperature conditions [1], but higher isoflavone concentration is accumulated at the seed maturing stage at low temperature [12, 13]. Light can also affect the isoflavone distribution and accumulation by adjusting the content and activity of key enzymes (such as PAL, CHS, CHI and IFS) in the isoflavone biosynthetic pathway [14, 15]. Isoflavone content decreased with increases in storage time and temperature, at the same time, the malonyl-glucoside components reach a high level [16–18].

Although several researches have studied soybean isoflavone content in a range of environments, few have investigated differences between cultivars of various maturity groups (i.e. 0-VI), which are grown in southern and northern China. In addition, few studies have related isoflavone content to other important seed composition characteristics, such as main quality traits and yield-related traits. The main purpose of this study was to determine the variation in isoflavone concentration among cultivars in various regions and years in China, and to analyze the relationship of isoflavone concentration to other seed characteristics and yield-related traits.

## Materials and Methods

### Plant Materials

Forty conventional soybean cultivars (Table 1), including three from the North region (NRT VER), twenty from the HuangHuaiHai valley region (HHH VER), fifteen from the South region (SRT VER), one from the USA, and one from Japan, selected from the Chinese soybean mini-core collections [19] were grown at two sites. The sample set of maturity group (MG) 0-VI consisted of two cultivars in MG 0, four in MG I, three in MG II, nine in MG III, fifteen in MG IV, four in MG V, and three in MG VI. There were two cultivars Amsoy (WDD00528-PI603373, USA) and Tokachi Nagaha (WDD01252-PI424209, Japan) from abroad. Soybean *cv.* Tokachi Nagaha introduced from Japan is one of the widely utilized elite soybean accessions in Chinese soybean breeding. Until 2005, 195 soybean cultivars released in China possess as their common parent *cv.* Tokachi-Nagaha [20]. Eleven cultivars are high protein types, in which protein content of *cv.* ZDD12680 is 51.8%, and two are high oil types, in which oil content of *cv.* ZDD07391 is 22.0 %. Other yield and quality traits are also different among these cultivars.

**Table 1:** Forty soybean cultivars from various ecotype regions and maturity groups

Ecotype region	Number	Cultivar identify code (maturity groups)
North region in China (NRT VER)	3	ZDD00041(0), ZDD00698 (I), ZDD07580 (I)
Huanghuaihai Valley region in China (HHH VER)	20	ZDD01761(III), ZDD02134 (III), ZDD02149 (V), ZDD02400 (IV), ZDD02764 (III), ZDD02891 (III), ZDD02892 (IV), ZDD02921 (III), ZDD03153 (IV), ZDD03570 (III), ZDD03741 (IV), ZDD04275 (IV), ZDD07391 (0), ZDD08190 (IV), ZDD09279 (IV), ZDD09884 (II), ZDD10100 (IV), ZDD18529 (II), ZDD18870 (IV), ZDD19027 (IV)
South region in China (SRT VER)	15	ZDD04620 (VI), ZDD05502 (I), ZDD06358 (III), ZDD06377 (IV), ZDD06378 (III), ZDD12527 (III), ZDD12680 (V), ZDD14125 (IV), ZDD14911 (V), ZDD15357 (IV), ZDD15624 (IV), ZDD16282 (V), ZDD20652 (V), ZDD21485 (VI), ZDD22145 (VI)
Abroad	2	WDD00528 (Amsoy, USA, II), WDD01252 (Tokachi Nagaha, Japan, I)

### Field Experiments

Seeds were planted at the Changping experimental station of Beijing (Site A, N 40°10′and E 116°14′) and Wuhan experimental station of Hubei province of China (Site B, N 30°29′and E 114°18′) in 2009 and 2010, located in two ecotopes of HHH VER and SRT VER, respectively. At the onset of experimentation, soil pH, all nitrogen, phosphorus, potassium and organic matter levels were 8.22, 80.5 mg kg^−1^, 68.7 mg kg^−1^, 14.58 g kg^−1^ and 12.31 g kg^−1^ at site A, respectively, and 7.69,51.27 mg kg^−1^, 13.56 mg kg^−1^, 113.95 g kg^−1^ and 19.83 g kg^−1^ at site B, respectively. The plots of each experiment were arranged in a randomized complete block design with three replications in a row length of 3 m, a row spacing of 0.5 m and plant spacing of 0.1 m. Plots were fertilized with 15 t ha^−1^ organic fertilizer, 30 kg ha^−1^of nitrogen and sufficient phosphorus and potassium during field preparation. Weeds were controlled by the post-emergence application of 2.55 L ha^−1^ of Acetochlor, as well as hand weeding later during the season. Plots were harvested by hand when the plants reached physiological maturity. As cultivars of different maturity groups were included, harvest data varied with cultivar. Seed moisture content was determined and yield and quality traits were expressed on a dry matter basis. Weather data for the growing season were retrieved in both years from a nearby weather station (Table 2).

**Table 2:** Monthly precipitation and average temperature in Beijing and Wuhan of China from April to September

Monthly	Precipitation (mm)				Temperature (°C)			
	Beijing		Wuhan		Beijing		Wuhan	
	2009	2010	2009	2010	2009	2010	2009	2010
April	63.6	32.2	54.3	197.7	15.8	15.9	18.5	18.3
May	64.1	14.7	344.2	132.1	20.3	22.9	24.7	22.2
June	125.3	95.5	129.4	306.7	23.4	26.2	29.3	28.1
July	79.3	196.6	148.1	95.9	27.2	27.0	26.4	30.3
August	132.1	60.9	240.7	38.8	26.0	25.7	29.3	29.1
September	118.9	23.3	40.8	41.8	21.0	21.1	28.4	24.9
Total/Mean	583.3	423.2	957.5	813.0	22.3	23.1	26.1	25.5

### Isoflavone Extraction

Samples from all plots were stored at room temperature after harvesting and extraction was within one month. Approximately 20 g of soybean seeds were ground using a cyclone mill (Retsch ZM100,Φ = 1.0 mm, Haan, Germany). Then, 0.1 g of powder was extracted using 5 ml 70% (V/V) ethanol solution containing 0.1% (V/V) acetic acid and shaken at room temperature for 12 h. After centrifugation (5,000 rpm, 5 min), the supernatant was filtered using a 0.22-μm nylon syringe filter (LUBITECH, Shanghai, China) and stored at 4°C for HPLC analysis.

### HPLC Assay

The isoflavone concentration was analyzed by the HPLC method [21]. First, 10 μL of the filtered extraction was subjected to High Performance Liquid Chromatography (HPLC) on an Agilent 1100 series system. Quantitative analyses were performed on the YMC Pack, ODS-AM-303 column (250 mm × 4.6 mm i.d., S-5 μm, 120 Å, YMC Co., Kyoto, Japan) at 35°C, using a 70-min linear gradient of 13-35% acetonitrile (v/v) in aqueous solution containing constant 0.1% acetic acid. The solvent flow rate was 1.0 mL min^−1^ and the UV absorption was measured at 260 nm. Twelve standards of isoflavone components, including daidzin (D), glycitin (GL), genistein (G), malonyldaidzin (MD), malonylglycitin (MGL), malonylgenistin (MG), acetyldaidzin (AD), acetylglycitin (AGL), acetylgenistin (AG), daidzein (DE), glycitein (GLE), and genistein (GE) were provided by Dr Akio Kikuchi (National Agricultural Research Center for Tohoku Region, Japan). Separate standard stock solutions were made for all of 12 isoflavone forms and stored at 4°C. According to the retention time and the maximum UV absorbance for 12 standards, we accurately detected all forms of isoflavone components based on the value of UV absorption at 260 nm. The various components of isoflavones, the aglycone and the total content in soybean seed were calculated from standard curves and expressed as micrograms per gram of dry weight according to the method described by Sun et al. [21].

### Soybean Agronomic Traits Measurement

Ten soybean plants of each cultivar per treatment were randomly selected to measure the agronomic traits, including plant height, bottom pod height, number of node, branch number, pods per plant, seeds per plant and 100-seed weight. The mean of ten plants per cultivar represented the replication value of agronomic traits and the average of three replications represented the value of agronomic traits.

### Protein and Oil Content Determination

A 50-g sample of soybean seeds was analyzed by Fourier transform near-infrared absorption spectroscopy (Bruker Fourier, Germany). The spectrum of each sample was the average of 3 replications, in which the absorption range was from 4000 to 8000 cm^−1^. The collected spectra were used to determine the protein and oil content by the Quant 2 method of Bruker’s OPUS 4.2 software. We took an average of 3 replications as the value of protein and oil content data.

### Fatty Acid Determination

The seed fatty acid content was determined using the gas chromatography methyl ester method [22]. First, 0.5 g of powder of soybean seeds were mixed with 1.5 mL hexane overnight, centrifuged at 7,000 rpm for 5 min, 350 μL of sodium methoxide solution was added, and then the mixture was shaken for 1 h. After centrifugation (7,000 rpm for 5 min) the supernatant was filtered into a special sample bottle for GC detectors. The GC analysis was performed on a RTX-Wax Column (30 m × 0.25 mm × 0.25 mm, Germany) with nitrogen, hydrogen and air as the carrier gas in 20 min. The injection volume was 1 μL. The area normalization method was used to calculate the percentage of five kinds of fatty acid compositions (palmitic acid, stearic acid, oleic acid, linoleic acid and linolenic acid) on a GC2010 workstation (Shimadzu, Japan).

### Statistical Analysis

All data were subjected to an analysis of variance (ANOVA) using the general linear model (GLM) procedure of the SAS software (SAS Institute, Cary, NC) to identify significant treatment effects and interactions. Homoscedasticity among the experiments was verified using the chi-square test. Data were then analyzed in a combined analysis that regrouped sites, maturity group, years and cultivars using PROC GLM. When interactions were significant, data were reanalyzed by sites and/or years. Comparisons between means were conducted with the least significant differences (LSD) at a 0.05 probability when ANOVA indicated model and treatment significances. Pearson’s *r* was calculated based on the data from all plots across the environments using the CORR procedure in SAS, to describe the relationship among the variables considered significant at *P* < 0.05.

## Results and Discussion

### Isoflavone Concentration Determination by HPLC

**Fig. 1:**
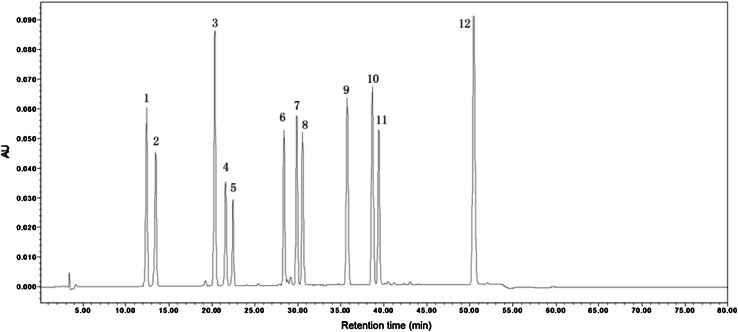
The HPLC chromatogram of the standard samples of 12 isoflavone components.* 1* daidzin,* 2* glycitin,* 3* genistin,* 4* malonyldaidzin,* 5* malonylglycitin,* 6* acetyldaidzin,* 7* acetylglycitin,* 8* malonylgenistin,* 9* daidzein,* 10* glycitein,* 11* acetylgenistin,* 12* genistein

This gradient elution method was rapid and accurate for determining and quantifying the amounts of 12 isoflavone components in soybean seeds by HPLC. The retention times and elution order of standard sample are shown in Fig. 1. In this study, six major isoflavone compositions, including daidzin (D), glycitin (GL), genistin (G), malonyldaidzin (MD), malonylglycitin (MGL), and malonylgenistin (MG), were detected in soybean seeds. Other components were not quantified due to their low concentrations in these soybean samples. It indicated that the glucoside and malonyl-glucoside groups were the main isoflavone components, while the free and acetyl-glucoside groups contents were too low to detect in soybean seeds. In this study, the total isoflavone content was described by the sum of these six isoflavone concentrations.

### Total Isoflavone Concentration in Soybean Cultivars

In this study, there was no significant difference among repeats in total isoflavone content, indicating the experimental data relative reliability. Cultivar (*P* < 0.001), site (*P* < 0.001) and year (*P* < 0.001) were the main effects and a (site × year × cultivar) interaction (*P* < 0.001) was observed for total isoflavone concentration (Table 3). The three-way interaction indicates that the magnitude of differences between cultivars and their ranking varied between environments (sites and years). However, the ranking of some cultivars with the highest and lowest concentrations was still relatively stable across environments based on the significant positive correlation between total isoflavone concentrations and years, sites (Table 4). Previous researchers reported similar results [23]. In our study, the coefficient of variation of total isoflavone concentrations ranged from 40.89% to 61.92%, corresponding to a total concentration ranging from 551.15 to 7584.07 μg g^−1^ and averaged 2972.64μg g^−1^ across environments (Table 5). There was a 45.01% variation in the average total isoflavone concentration across environments and this variation ranged between 19.67% and 68.87% for special cultivars. Total isoflavone content also varied among various years and sites (*P* < 0.001) in this study. Previous studies showed that the accumulation of isoflavone was greatly influenced by temperature, rainfall and other climate factors in soybean seeds [23, 24]. In our studies, in two growing seasons, the average concentration of total isoflavone in 2010 was 87.83% higher than in 2009; Furthermore, in two planting sites, total isoflavone content at Beijing was also 71.96% higher than at Wuhan (Table 6). Indeed, the precipitation and temperature was different in the planting seasons of 2009 and 2010 at both sites. In Beijing, precipitation in 2009 was 160.01 mm higher than in 2010; in Wuhan, precipitation in 2009 was also 144.5 mm higher than in 2010. Moreover, at both sites, precipitation and average temperature in Wuhan were significant higher than in Beijing (Table 2). We confirmed that high temperature and moisture stress during seed-fill can reduce total isoflavone accumulation in soybean seeds, corresponding to the previous results [12]. This underlines the fact that climatic conditions have an important impact on total isoflavone concentration.

**Table 3:** Analysis of variance for the isoflavone concentrations of soybean cultivars grown at Beijing and Hubei of China in 2009 and 2010

Variance sources	Isoflavone components							
	*df*	Daidzin	Glycitin	Genistin	Malonyldaidzin	Malonylglycitin	Malonylgenistin	Total content
Maturity group	6	***	***	**	**	***	*	**
Year	1	***	***	***	*	*	**	***
Site	1	NS	***	**	***	*	***	***
Site × Year	1	***	NS	**	**	NS	NS	**
Repeat	2	**	NS	**	NS	NS	NS	NS
Cultivar	39	***	***	***	***	***	***	***
Cultivar × Site	39	***	***	***	***	***	***	***
Cultivar × Year	39	NS	**	*	***	**	**	***
Cultivar × Site × Year	39	**	***	*	***	***	*	***

*, ** and *** represent the significance levels at *P* ≤ 0.05, 0.01, and 0.001, respectively; *NS* not significant

**Table 4:** Correlation analysis between years or sites on the isoflavone components in soybean seeds

Environmental factor	Daidzin	Glycitin	Genistin	Malonylglycitin	Malonylgenistin	Malonylglycitin	Total content
Year^†^	0.743**	0.153	0.604**	0.744**	0.549**	0.597**	0.704**
Site^§^	0.533**	0.242	0.522**	0.499**	0.563**	0.508**	0.460**

** represents the significant level at *P* ≤ 0.01

^†^ Correlation coefficient between two years on mean isoflavone contents were measured based on the two-site data

^§^Correlation coefficient between two sites on mean isoflavone contents were measured based on the two-year data

**Table 5:** Genetic variation analysis of the isoflavone components in soybean seeds^†^

Isoflavone component	Range	Minimum (μg g^−1^)	Maximum (μg g^−1^)	Mean (μg g^−1^)	Std. Deviation	CV%
Daidzin	1218.15	31.09	1249.24	251.64	175.17	69.61
Glycitin	491.62	0.00	491.62	108.25	66.32	61.27
Genistin	1452.69	40.37	1493.05	465.78	272.11	58.42
Malonyldaidzin	2322.01	32.64	2354.65	707.00	407.77	57.68
Malonylglycitin	676.08	0.00	676.08	135.17	69.96	51.76
Malonylgenistin	3473.66	189.48	3663.14	1265.78	576.83	45.57
Total content	7032.92	551.15	7584.07	2972.64	1337.84	45.01

*CV * coefficient of variation

^†^ The isoflavone values for individual components and total contents were measured based on the two-site and two -year collected data

**Table 6:** Comparison of isoflavone composition in various years and sites in soybean seeds

Isoflavone component	Year^†^	Mean (μg g^−1^)	Std. Deviation	Site^§^	Mean (μg g^−1^)	Std. Deviation
Daidzin	2009	112.42	55.92	Beijing	352.07	169.49
	2010	352.07	169.49	Wuhan	276.52	171.91
Glycitin	2009	63.54	37.54	Beijing	100.30	49.35
	2010	100.30	49.35	Wuhan	144.89	75.06
Genistin	2009	200.56	90.39	Beijing	638.50	213.68
	2010	638.50	213.68	Wuhan	533.97	253.48
Malonyldaidzin	2009	638.70	258.60	Beijing	1058.29	379.24
	2010	1058.29	379.24	Wuhan	417.74	262.04
Malonylglycitin	2009	122.90	73.58	Beijing	158.28	65.50
	2010	158.28	65.50	Wuhan	123.19	65.49
Malonylgenistin	2009	1108.12	384.39	Beijing	1767.84	487.24
	2010	1767.84	487.24	Wuhan	906.93	441.30
Total content	2009	2228.84	710.98	Beijing	4186.43	1123.95
	2010	4186.43	1123.95	Wuhan	2434.47	1107.97

^†^Mean isoflavone contents in various years were measured based on the two-site data

^§^ Mean isoflavone contents in various sites were measured based on the two-year data

Significant differences in total isoflavone content were also observed among maturity groups (Table 7). Maturity groups V and VI had significantly higher total isoflavones when compared to maturity groups 0-IV. However, the differences among MG 0-IV and MG V-VI were not significant in total isoflavone contents. There was a similar trend on total isoflavone content, where lower isoflavone concentrations were generally presented in early rather than late maturing soybean cultivars. For example, the average total isoflavone content in MG 0 varieties (2225.21μg g^−1^) was the lowest, while in MG VI (3569.41μg g^−1^) it was higher than in the other maturity groups. Especially the total content in MG VI was 60.41% higher than in MG 0.

**Table 7:** Comparison of isoflavone composition among maturity groups^§^ in soybean seeds^†^

Maturity group	Daidzin	Glycitin	Genistin	Malonyldaidzin	Malonylglycitin	Malonylgenistin	Total content
0	182.13^a^	67.42^a^	351.61^a^	501.19^a^	87.60^a^	1018.98^a^	2225.21^a^
I	247.39^a^	89.37^ab^	373.89^a^	613.82^a^	108.79^a^	977.86^a^	2473.48^a^
II	231.91^a^	107.27^b^	433.28^a^	598.16^a^	114.88^b^	1112.75^a^	2620.57^a^
III	246.27^a^	111.87^b^	445.29^a^	697.53^a^	131.12^b^	1204.71^a^	2878.25^a^
IV	203.23^a^	110.59^b^	410.31^a^	597.37^a^	137.04^b^	1125.61^a^	2616.37^a^
V	338.94^b^	104.17^ab^	557.32^b^	830.01^b^	132.89^b^	1349.20^b^	3351.17^b^
VI	337.87^b^	158.14^c^	581.47^b^	818.16^b^	180.78^c^	1445.87^b^	3569.41^b^

^§^ Isoflavone contents are expressed in μg g^−1^

^†^ Mean values within a column, in each maturity group followed by the same letter are not significantly different at the 0.05% level as determined by Fishers LSD test. Mean isoflavone contents for each maturity group were measured based on the two-site and two-year data

Few previous studies considered the maturity group as a variable. According to our results, it is evident that the isoflavone composition of samples varies among maturity groups, corresponding to the results of Wang et al., [24]. The underlying mechanism for these observations might be attributable to climatic conditions and an isoflavone accumulation pattern, which can explained that in the same planting site, more late maturing cultivars have longer growing stages and accumulate more isoflavone in their seeds [25]. Moreover, lower temperatures occurred at the R7-8 stages of late maturing cultivars, which can also increase seed isoflavone content [12].

Previous studies have reported that isoflavone concentrations in soybean seeds, as a quantitative trait, was controlled by both genetic and environmental factors [5, 6, 8–11, 24]. Our results showing significant differences for individual and total isoflavone contents among cultivars also confirmed that genetic factors play an important role in soybean isoflavone breeding. However, there were also significant differences among years and plots, indicating that environmental factors could not be ignored with regard to isoflavone production in soybean seeds. Especially, the factor of year should be given more attention. Therefore, selection for isoflavone components in a single environment within a single year is not likely to be effective [11, 26, 27].

### Individual Isoflavone Concentrations in Soybean Cultivars

Across environments, daidzin, glycitin, genistin, malonyldaidzin, malonylglycitin and malonylgenistin represented 9.46%, 3.69%, 17.66%, 22.32%, 4.25% and 40.45% of the total isoflavones, respectively. Moreover, the malonyl-glucoside groups, including malonyldaidzin, malonylglycitin and malonylgenistin, accounted for 67.01% of the total isoflavone content (Table 5]. It indicated that the malonyl-glucoside groups play an important role with regard to isoflavone components in soybean seeds. Previous studies have demonstrated that there was the abundant genetic variation effect on the isoflavone content. Especially, the malonyl-glucoside group in the isoflavone composition plays an important role in the soybean seed [1, 9, 10, 13, 28]. In our studies, we confirmed that soybean breeding selection for isoflavones should mainly focus on the isoflavone malonyl-glucoside group.

The proportions in the total concentrations of individual isoflavones also varied among cultivars (*P* < 0.001). Responses to site, year, cultivar and their interactions also differed for each of the individual isoflavones (Table 3). For example, the daidzin concentration varied among cultivars (*P* < 0.001), site (*P* < 0.01) and year (*P* < 0.001), while not interacting with the year. As for total isoflavones, the daidzin concentration ranged from 31.09 to 1249.24 μg g^−1^, averaging 251.64 μg g^−1^ across environments (Table 5). A site × year interaction (*P* < 0.001) was also observed as daidzin concentrations were greater at Beijing in 2010 than any of the other environments (Table 3). Genistin concentration was also affected by the cultivar (*P* < 0.001), which interacted with site (*P* < 0.001) and year (*P* < 0.01). This interaction was attributable to a generally greater genistein concentration in most cultivars, but to different degrees at Beijing compared to Wuhan. Across sites and cultivars, genistin concentration was also higher in 2010 than 2009 (Table 6). Other isoflavones, such as glycitin, malonyldaidzin, malonylglycitin and malonylgenistin also had differences in concentrations among cultivars (*P* < 0.001), year (*P* < 0.001) and site (*P* < 0.001). However, no significant interactions of site × year for glycitin, malonylglycitin and malonylgenistin were presented in this study. Finally, the three-way interactions were observed for all individual isoflavone compositions in spite of various significant levels, indicating that concentrations for cultivars and their ranking varied greatly among environments.

As for total isoflavone content, significant differences in individual isoflavone concentrations were also observed among maturity groups. In general, there was also a similar trend on individual isoflavone contents, where lower isoflavone concentrations were generally presented in early rather than late maturing soybean cultivars. Especially, glycitin and malonylglycitin concentrations in MG 0 were significantly lower than in MG II-IV (Table 7), indicating that glycitin and malonylglycitin concentrations are more sensitive to the day of maturity. Our results are in agreement with those of Wang et al. [24], who reported a positive correlation between daidzin and days of maturity, however, they also reported negative correlations between days of maturity and genistin, daidzein and genistein concentrations. In contrast, Seguin et al. [23] reported no clear differences between maturity groups and individual isoflavone concentrations.

### Relationship Between Individual and Total Isoflavones

Individual isoflavone components had highly significant positive correlations with the total isoflavone content (Table 8). The highest (*r* = 0.925**) and lowest (*r* = 0.406**) correlation coefficients was shown between total content and malonylgenistin, between total content and malonylglycitin, respectively, which indicated that malonylgenistin is the main contributor to total isoflavone concentration. There were statistically significant positive correlations of aglycone with their corresponding components and total aglycone content (data not shown), which indicated that the increases in glucoside components, including daidzin, glycitin and genistin, accumulation caused the malonyl-glucoside group, including malonyldaidzin, malonylglycitin and malonylgenistin, to increase correspondingly. These correlations are not surprising as individual isoflavones are synthesized via a common phenylpropanoid pathway [14, 29].

**Table 8:** Correlation analysis among the isoflavone components in soybean seeds^†^

Isoflavone composition	Daidzin	Glycitin	Genistin	Malonyldaidzin	Malonylglycitin	Malonylgenistin
Glycitin	0.479**					
Genistin	0.912**	0.520**				
Malonyldaidzin	0.726**	0.293**	0.702**			
Malonylglycitin	0.159*	0.461**	0.238**	0.338**		
Malonylgenistin	0.567**	0.268**	0.737**	0.791**	0.376**	
Total content	0.812**	0.447**	0.892**	0.907**	0.406**	0.925**

* and ** represent significance levels at *P* ≤ 0.05 and 0.01, respectively

^†^ Correlation coefficient between mean isoflavone contents were measured based on the two-site and two-year data

### Correlations Between Isoflavone Concentrations and Other Seed Characteristics

In this study, highly significant correlations were observed between both years and between both sites for individual and total isoflavone contents in this study (Table 4), indicating that the ranking of cultivars with the highest and lowest isoflavone concentrations across years and sites was relatively stable. It also demonstrated that genetic factors still play an important role in isoflavone content although this is significantly affected by the environments.

Significantly positive correlations between total and individual isoflavones and seed yield-related traits were observed, e.g., for total isoflavone correlations, plant height (*r* = 0.488**), effective branches (*r* = 0.398**), pods per plant (*r* = 0.364**), and seeds per plant (*r* = 0.302**) (Table 9), indicated that high-yield and taller plant varieties tend to have higher total isoflavone concentrations.

**Table 9:** Correlations between isoflavone contents and seed yield related traits, quality traits^†^

Trait	Daidzin	Glycitin	Genistin	Malonyldaidzin	Malonylglycitin	Malonylgenistin	Total content
Palmitic acid	−0.027	0.346^**^	−0.058	0.040	0.282^**^	−0.014	0.024
Stearic acid	−0.055	−0.310^**^	0.013	−0.078	−0.246^*^	0.000	−0.053
Oleic acid	−0.366^**^	−0.301^**^	−0.376^**^	−0.403^**^	−0.403^**^	−0.398^**^	−0.427^**^
Linoleic acid	0.448^**^	0.150	0.481^**^	0.485^**^	0.278^**^	0.470^**^	0.493^**^
Linolenic acid	0.269^**^	0.259^*^	0.261^*^	0.279^**^	0.331^**^	0.281^**^	0.306^**^
Oil content	−0.317^**^	−0.260^*^	−0.264^**^	−0.392^**^	−0.348^**^	−0.288^**^	−0.344^**^
Protein content	0.122	0.187	0.039	0.137	0.243^*^	0.054	0.100
Plant height	0.357^**^	0.351^**^	0.425^**^	0.475^**^	0.384^**^	0.463^**^	0.488^**^
Bottom pod height	0.147	−0.172	0.131	0.177	0.001	0.136	0.145
Node number	0.155	0.044	0.158	0.161	0.044	0.111	0.141
Effective branch	0.318^**^	0.139	0.322^**^	0.435^**^	0.225^*^	0.372^**^	0.398^**^
Pods per plant	0.327^**^	0.276^**^	0.362^**^	0.340^**^	0.246^*^	0.335^**^	0.364^**^
Seeds per plant	0.246^*^	0.283^**^	0.299^**^	0.265^**^	0.223^*^	0.288^**^	0.302^**^
100-seed weight	−0.021	−0.042	0.017	−0.017	−0.027	0.038	0.012

* and ** represent the significant levels at *P* ≤ 0.05 and 0.01, respectively

^†^ Correlation coefficient between mean isoflavone contents and agronomic or quality traits were measured based on the two-site and two-year data

For seed quality traits, significant positive correlations between total isoflavone and linoleic acid (*r* = 0.493**) and linolenic acid (*r* = 0.306**) were observed. However, significant negative correlations were observed between total isoflavones and oleic acid (*r* = −0.427**) and oil content (*r* = −0.344**). It indicated that oil accumulation and fatty acid compositions can affect isoflavone concentration in soybean seeds. Furthermore, significant positive correlations were also observed between palmitic acid and glycitin (*r* = 0.346**), malonylglycitin (*r* = 0.282**); while negative correlations were observed between stearic acid and glycitin (*r* = −0.310**), malonylglycitin (*r* = −0.246*). However, correlations between palmitic acid, stearic acid and other isoflavones were insignificant (Table 9). It indicated that both of palmitic acid and stearic acid were poor indicators of the isoflavone content of the sample.

Previous studies reported that protein content had significant negative correlations with isoflavone content [5, 23], however, in our study, for protein content, a weak, but significant positive correlation with malonylglycitin (*r* = 0.243*) was observed, while there were no significant correlations with other isoflavones (Table 9). In contrast, with regard to oil content, significant negative correlations with individual isoflavones were also observed.

Our results are in agreement with those of Seguin et al. [23], Wang et al. [24] and Vyn et al. [30], who also reported positive correlations between seed yield related traits and total and several individual isoflavones. However, Wang et al. [24] also reported negative correlations between seed yield and genistein, between plant height and genistin, malonyldaidzin. Furthermore, Primomo et al. [6] reported that isoflavone shared a common locus with plant height on Chromosome 5. Moreover, seed oil content also shared one genomic region with genistein on Chromosome 5 and glycitein on Chromosome 3, whereas seed weight shared regions with these two isoflavones on Chromosome 5, 6, and 13. The previous studies also verified our correlation results on the genomic level. Therefore, we can predict that plant height may be a better indicator of isoflavone content in some environments.

## Conclusions

This study suggests that environmental factors have a great impact on seed isoflavone contents of various maturity soybean cultivars, as indicated by significant site, year and site × year × cultivar effects. There was a similar trend on the individual and total isoflavone contents, in which lower isoflavone concentrations are generally presented in early rather than late maturing soybean cultivars. However, cultivars with consistently high or low isoflavone concentrations across environments were identified in spite of significant cultivar × year × site interactions, demonstrating that the genetic factor plays the most important role for isoflavone accumulation. The positive correlations we observed between total isoflavone concentration and plant height, effective branches, pods per plant, seeds per plant, linoleic acid and linolenic acid, plus negative correlations with oleic acid and oil content, indicating that isoflavone concentration can be predicted as being associated with other desirable seed characteristics.
